# Electrically switchable metallic polymer metasurface device with gel polymer electrolyte

**DOI:** 10.1515/nanoph-2022-0654

**Published:** 2023-01-24

**Authors:** Derek de Jong, Julian Karst, Dominik Ludescher, Moritz Floess, Sophia Moell, Klaus Dirnberger, Mario Hentschel, Sabine Ludwigs, Paul V. Braun, Harald Giessen

**Affiliations:** 4th Physics Institute and Research Center ScoPE, University of Stuttgart, Pfaffenwaldring 57, 70569 Stuttgart, Germany; Department of Materials Science and Engineering, Materials Research Laboratory, and Beckman Institute for Advanced Science and Technology, University of Illinois Urbana-Champaign, Urbana, IL 61801, USA; IPOC-Functional Polymers, Institute of Polymer Chemistry, University of Stuttgart, Pfaffenwaldring 55, 70569 Stuttgart, Germany

**Keywords:** beam switching, metadevice, metasurfaces, nanophotonics, plasmonics, polymers

## Abstract

We present an electrically switchable, compact metasurface device based on the metallic polymer PEDOT:PSS in combination with a gel polymer electrolyte. Applying square-wave voltages, we can reversibly switch the PEDOT:PSS from dielectric to metallic. Using this concept, we demonstrate a compact, standalone, and CMOS compatible metadevice. It allows for electrically controlled ON and OFF switching of plasmonic resonances in the 2–3 µm wavelength range, as well as electrically controlled beam switching at angles up to 10°. Furthermore, switching frequencies of up to 10 Hz, with oxidation times as fast as 42 ms and reduction times of 57 ms, are demonstrated. Our work provides the basis towards solid state switchable metasurfaces, ultimately leading to submicrometer-pixel spatial light modulators and hence switchable holographic devices.

## 1 Introduction

Metasurfaces show tremendous potential in optical devices for technologies including virtual reality (VR) [1], augmented reality (AR) [2], [3], [4], and autonomous driving (LiDAR systems) [5, 6]. In principle, metasurfaces can generate arbitrary wavefronts to mimic and realize lenses [7], [8], [9], [10], beam deflectors [11], [12], [13], holograms [14, 15], and many other optical functionalities [16], [17], [18]. Most importantly, their integration and combination with on-chip electronic (and optical) circuits will allow significant miniaturization of optical devices.

What is more, by combining metasurfaces with active materials, unique and novel functionalities become possible as the optical properties can be dynamically adjusted [19], [20], [21], [22]. In such way, active metalenses [23], [24], [25], [26], [27], [28], [29], [30], [31], beam switching [25, 32], [33], [34] and steering devices [6, 35], [36], [37], active holograms [38, 39], dynamic displays [40], [41], [42], or spatial light modulators [43] have been demonstrated. Recently, a new concept for active nanoantennas and metasurfaces based on polymers has been introduced [23, 33, 44], [45], [46]. In particular, metasurfaces made from metallic polymers allow one to reversibly switch plasmonic resonances fully ON and OFF via an applied voltage [23, 33]. The comprising nanoantennas are electrically switchable between metallic and insulating states at display frequencies and CMOS (complementary metal-oxide-semiconductor) compatible voltages. The fabricated metasurfaces can be operated in transmission with 100% switching contrast. Besides using liquid electrolytes for the electrochemical switching process, gel electrolytes have also proven to be suitable to switch plasmonic resonances of metallic polymer nanoantennas [45]. Yet, so far, only liquid electrolytes in combination with metallic polymer metasurfaces have demonstrated functionalities such as beam switching with full contrast and fast switching frequencies [23, 33].

Here, we combine a metallic polymer metasurfaces with a gel polymer electrolyte to realize a polymer metadevice for electro-active beam switching. The standalone device has a very small footprint, can be operated at CMOS compatible voltages, and shows switching frequencies as high as 10 Hz. Due to the gel electrolyte approach, our work presents an important step towards the integration of active metallic polymer nanoantennas and metasurfaces into optical devices. With our approach, on-chip pixel sizes below 1 µm of future spatial light modulators can become possible, which are a key element for future optical technologies including 3D holography.

## 2 Results and discussion

**Figure 1:**
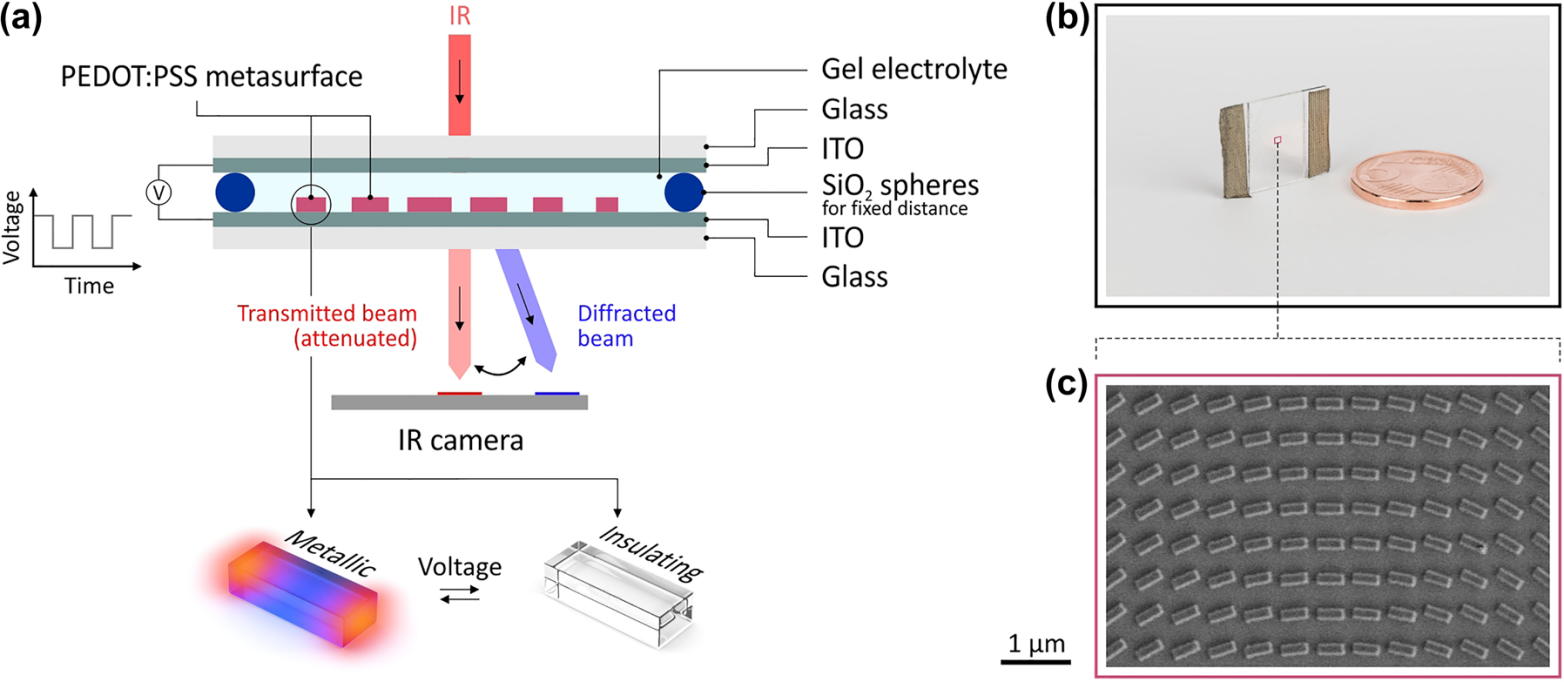
Metallic polymer metadevice for beam switching. (a) Schematic concept of metadevice based on gel electrolyte. A metasurface from metallic polymer PEDOT:PSS is fabricated on an ITO-covered glass substrate. The metasurface is embedded in a gel polymer electrolyte (LiClO_4_/PEO/acetonitrile). An ITO covered glass seals the device from the top. SiO_2_ spheres (diameter 10 µm) are used to control the thickness of the electrolyte. A voltage applied between the two ITO slides switches the polymer metasurface and the individual nanoantennas reversibly between metallic (plasmonic) and insulating state. This allows for beam switching, where the diffracted beam can be turned ON or OFF. (b) Photograph of the metadevice compared to the size of a five cent coin. The metasurface is marked in red. (c) SEM image of the beam switching metasurface comprising metallic polymer nanoantennas. The individual nanoantennas have dimensions of length = 380 nm, width = 160 nm, and height = 90 nm. The superperiod is 15 µm.

The concept of our metadevice is illustrated in Figure 1a. The metasurface for beam switching is made from the metallic polymer poly(3,4-ethylenedioxythiophene):poly(-styrene sulfonate) (PEDOT:PSS) and is fabricated via an electron beam lithography and dry etching process on an indium tin oxide (ITO) covered glass substrate. ITO is used as electrode for electrical access to the nanoantennas. The nanoantennas are embedded in a gel electrolyte composed of LiClO_4_ in polyethylene oxide (PEO) and acetonitrile. Another ITO covered glass substrate seals the metadevice from the top. To control the exact thickness of the electrolyte and thus the spacing between the two ITO electrodes, silicon dioxide (SiO_2_) spheres with a diameter of 10 µm are used. They are mixed into the electrolyte and thus determine the thickness when pressing the cell during assembly. Please refer to Figure S1 in the Supporting Information for details on the exact mixture of our gel polymer electrolyte and for further information on the fabrication process.

As mentioned, the metasurface consists of electrically switchable polymer nanoantennas. Depending on the applied voltage between the two ITO electrodes, the nanoantennas can either be switched into a metallic plasmonic state (positive voltage) or into an insulating state (negative voltage). This is depicted in the lower part of Figure 1a. The reason is a drastic change of the doping state and the charge carrier density in the polymer upon the electrochemical redox reaction which results in a tuning and shift of the plasma frequency. A positive voltage fully dopes the polymer, leading to a high free charge carrier density and metallic properties. In contrast, a negative voltage causes a de-doping of the polymer, low charge carrier densities, and insulating optical properties.

To realize the active beam switching metasurface we use a geometrical phase approach. Hereby, neighboring nanoantennas are subsequently rotated by an angle of 6° – equivalent to a full rotation after 30 nanoantennas and a superperiod of 15 µm. At an illumination wavelength of *λ* = 2.65 µm, this superperiod corresponds to a diffraction angle of 10.2°. Consequently, an impinging circularly polarized light beam is diffracted depending on the state of the polymer. The diffracted beam switches ON for metallic polymer properties, whereas it is switched OFF when the polymer turns insulating.

A photograph of the entire metadevice is depicted in Figure 1b. The lateral size is comparable to the size of a 5 cent coin. We use metal electrode tape to ease clamping of the electrodes. The actual active metasurface area is marked in red. A corresponding scanning electron microscope (SEM) image of the polymer nanoantennas and metasurface prior to the cell assembly is shown in Figure 1c, illustrating the excellent structure quality.

**Figure 2:**
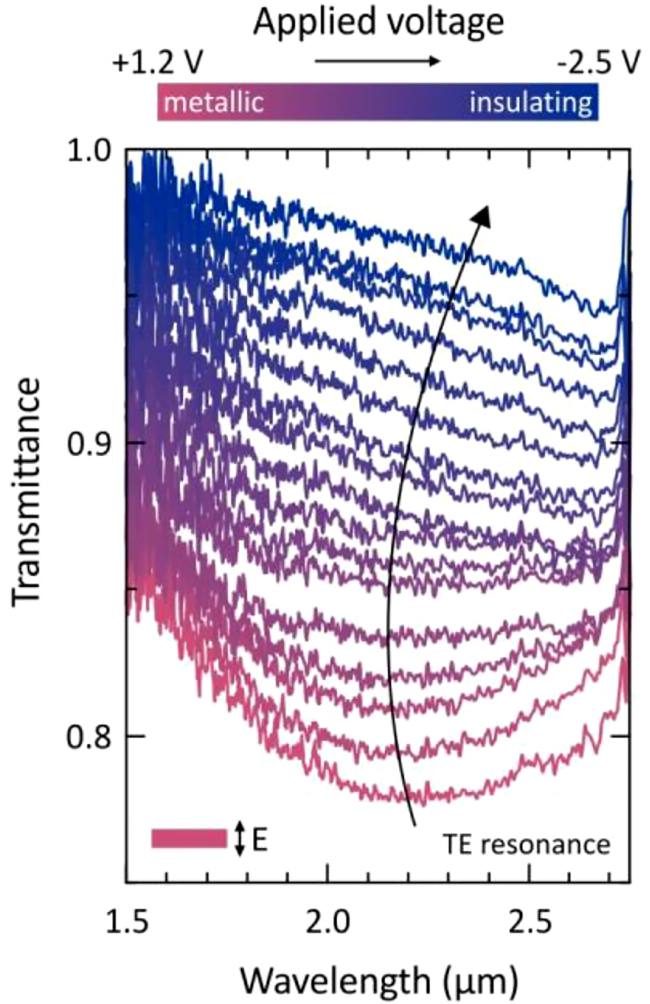
Electrically switchable plasmonic resonance. Plasmonic resonance of polymer nanoantennas (transverse electric field) in the infrared spectral range for different applied voltages. The voltage is changed from +1.2 V (polymer fully metallic with plasmonic resonance ON) to −2.5 V (polymer insulating with plasmonic resonance OFF). The resonance modulation vanishes with decreasing voltage until the plasmonic resonance has been switched off completely.

When applying a voltage between the two ITO electrodes, we can switch the plasmonic nanoantennas ON or OFF. This is demonstrated in Figure 2. We use a Fourier-transform-infrared-spectrometer (FTIR) to measure the transmittance through the metadevice/metasurface as a function of wavelength in the infrared spectral range, while the voltage is changed from +1.2 V to −2.5 V. One can see that for a positive voltage of +1.2 V the plasmonic resonance is ON (red curve). The polymer nanoantennas are fully metallic with highest charge carrier density. The TE (transverse electric) plasmonic resonance (electric field along the short axis of the nanoantennas) possesses highest modulation of 22% in transmission at a wavelength of *λ* = 2.2 µm. Please note that the polymer needs to be intrinsically lossy to realize a plasmonic resonance. This loss/absorption can be attributed to the imaginary part *ε*_2_ of the dielectric function [33]. When slowly changing the voltage from +1.2 V to −2.5 V (going from red to blue in Figure 2), the charge carrier concentration in the polymer nanoantennas is reduced, resulting in a gradual decrease of the amplitude of the plasmonic resonance. Finally, at a voltage of −2.5 V, the polymer is switched into the fully reduced and insulating state. The nanoantennas now possess *no* plasmonic resonance. This process is fully reversible as will be demonstrated below by analyzing the beam switching metasurface in detail.

**Figure 3:**
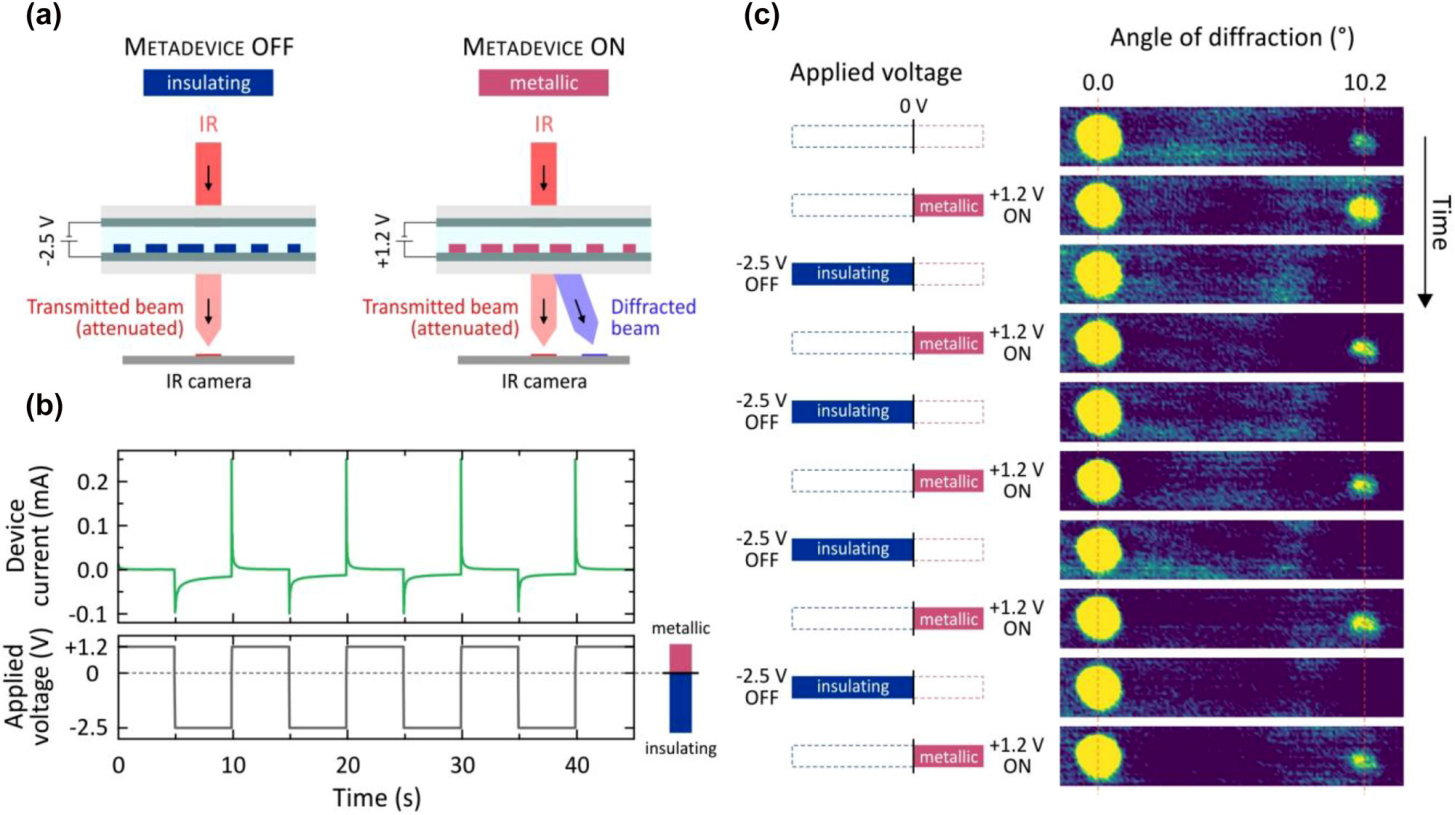
Active beam switching with metallic polymer metadevice. (a) States of the gel-based beam switching metadevice. Right: an applied voltage of +1.2 V turns the metasurface into the metallic state, the metadevice is ON and diffracts the incoming IR beam (wavelength *λ* = 2.65 µm). Left: an applied voltage of −2.5 V turns the metasurface insulating and the metadevice OFF with no diffraction. (b) Bottom: applied voltage as function of time oscillating between +1.2 V (metallic) and −2.5 V (insulating). Measured current flowing through the device as function of time when the applied voltage is changed. It shows fast saturation of the current, demonstrating fast oxidation and reduction times of the polymer. (c) Measured camera images while the voltage is changed between +1.2 V and −2.5 V. The diffracted beam at 10.2° can be subsequently switched ON and OFF depending on the applied voltage. The contrast of the intensity in the diffracted beam of the metadevice between ON and OFF state is 100%.

The functionality and possible states of our beam switching metasurface are illustrated in Figure 3a. An applied voltage of +1.2 V turns the metadevice ON, diffracting the incoming circularly polarized light beam (*λ* = 2.65 µm) to an angle of 10.2° (right sketch). One will observe the diffracted (1st order) as well as the purely transmitted (0^th^ order) beam. Please note that the transmitted beam is attenuated to prevent saturation of the infrared camera. An applied voltage of −2.5 V turns the metadevice OFF, the nanoantennas become insulating with no diffracted beam observed (left sketch).

We now apply a square wave voltage changing between +1.2 V and −2.5 V as a function of time as shown in Figure 3b (lower graph). Each voltage is applied for 5 s each, while we simultaneously measure the current flowing through the device (upper graph, green curve). A total of 4.5 switching cycles are presented. One can see that the device current follows the change of the applied voltage. The current quickly saturates, demonstrating fast and full oxidation as well as reduction of the polymer nanoantennas. At the same time, we capture the intensities of the transmitted (0^th^ order) and the diffracted (1st order) beam with the IR camera (details on the exact measurement setup are shown in Figure S2a in the Supporting Information). The results are depicted in Figure 3c. The first image (top) illustrates the pristine state of the metadevice when no voltage (0 V) is applied. For this voltage, the polymer is already metallic, yet, not fully doped. Thus, the charge carrier density is not at its maximum and only a small fraction of the incoming light is diffracted to 10.2° by the metasurface. When the applied voltage is switched to +1.2 V, the polymer nanoantennas are doped and become metallic. The metadevice is switched fully ON and we observe the maximum diffracted intensity. Please note that, so far, the diffraction efficiency in this state is around 1.1%. Yet, further improvement could be achieved in the future by, e.g., increasing the plasmonic resonance modulation in the metallic state. In contrast, the diffracted beam vanishes on the IR camera, when the applied voltage of −2.5 V switches the nanoantennas into the insulating state and thus the metadevice OFF. As expected, the data illustrates that the diffracted beam is switched with a contrast of 100%. To further illustrate the device functionality, we show in Figure 3c 4.5 cycles of switching, demonstrating reversible ON and OFF switching of our metadevice. The intensity of the diffracted beam decreases for subsequent cycles, which is mostly due to degradation of the polymer metasurface. The degradation and switching contrast of the beam switching metallic polymer metasurfaces should be highly influenced by the exact covering of the nanoantennas with the gel electrolyte. Consequently, factors such as electrolyte deposition or device assembly might lead to significant improvements.

**Figure 4:**
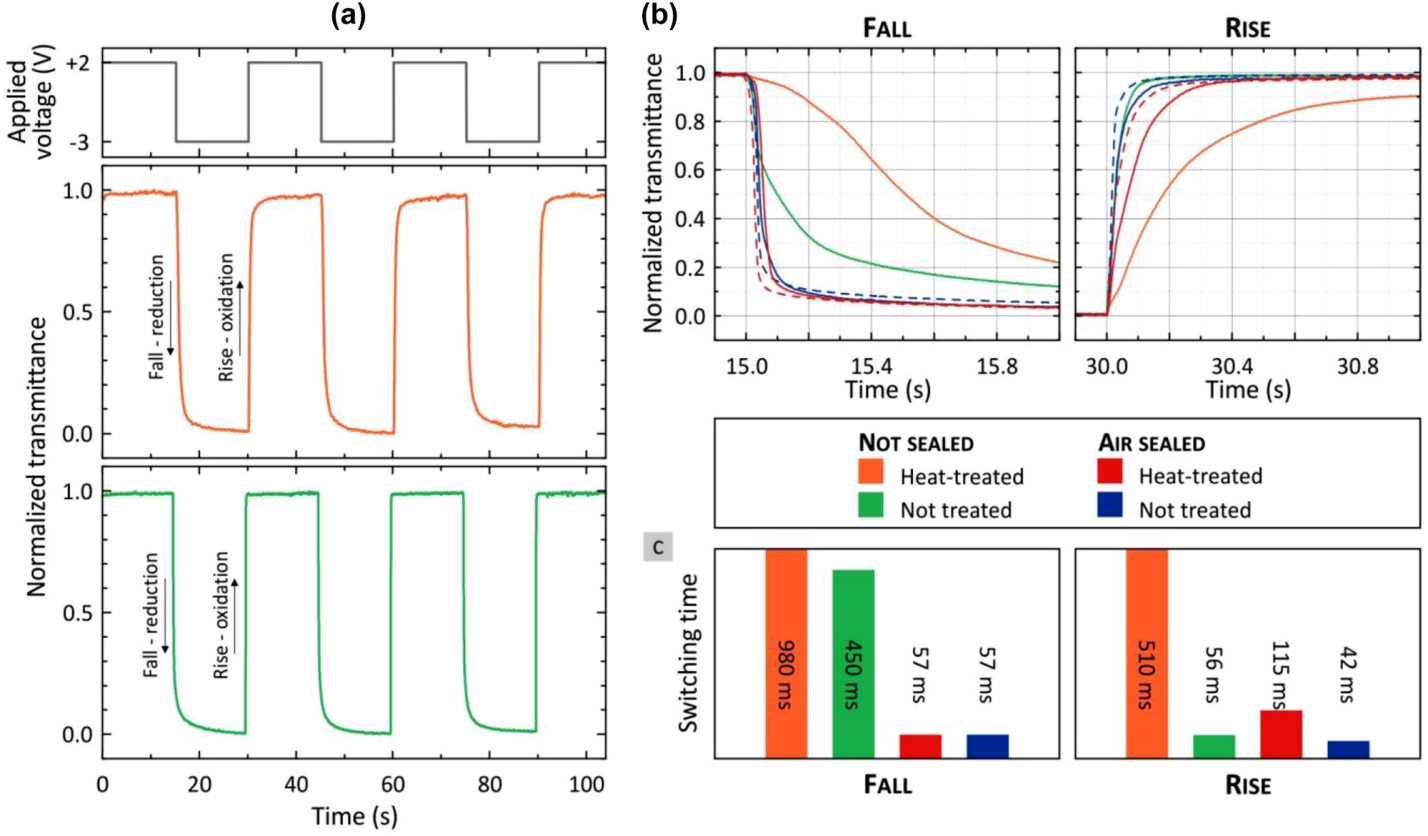
Switching speed for differently fabricated PEDOT:PSS film devices. (a) Normalized transmittance as function of time while the applied voltage (top graph) is switched for differently prepared PEDOT:PSS films. The middle graph (orange curve) depicts the response for PEDOT:PSS which was heat-treated (120 °C for 15 min) after spin-coating and before the cell assembly. The bottom graph (green curve) shows the response when no heat-treatment of PEDOT:PSS was performed (drying at room temperature). In both cases, the device was not air-sealed. (b) Zoom-in on first fall curves (left) and rise curves (right) of switching for differently prepared devices. Orange: PEDOT:PSS heat-treated, device not sealed. Green: PEDOT:PSS not treated, device not sealed. Red: PEDOT:PSS heat-treated, device air-sealed (2 samples, solid and dashed). Blue: PEDOT:PSS not treated, device air-sealed (2 samples, solid and dashed). (c) Extracted fall and rise times from (b). One finds significantly faster switching times for devices which have been air-sealed, where switching frequencies of 10 Hz are possible. Additionally, the rise times become even faster for PEDOT:PSS which was not heat-treated.

A crucial parameter for the applicability of our metadevice with gel polymer electrolyte in future technologies is its switching speed. We investigate the switching time constants of our metallic polymer in combination with the gel electrolyte in Figure 4. The switching time is dependent on the choice of the conducting polymer and the electrolyte, which is needed for charge balancing during an electrochemical experiment: Upon doping and de-doping counterions are reversibly incorporated and expelled from the bulk of the conducting polymer film to provide charge electroneutrality [47]. Hereby, we use thin films of PEDOT:PSS, as they allow to carry out switching measurements in the visible spectral range with large signal to noise ratio and excellent time resolution. The sample is illuminated with a helium neon laser and the transmitted intensity is detected with a photodiode while the state of the PEDOT:PSS film is controlled via the externally applied voltage. Note that we directly read out unreferenced voltages from the photodiode and thus plot normalized transmittance values. Please refer to Figure S2b in the Supporting Information for a sketch of the exact measurement setup. In particular, we compare in Figure 4a the switching of two differently fabricated 90 nm thin films of PEDOT:PSS. One film was heat-treated after spin coating (orange) at a temperature of 120 °C for 15 min. This is the standard drying procedure during the fabrication of PEDOT:PSS nanoantennas shown above and in our previous work [23, 33]. Please note that the heat treatment was performed prior to device assembly without heating the electrolyte. The other PEDOT:PSS film was *not* heat-treated and instead dried at room temperature (green). Both devices are not sealed and thus not protected from oxygen in the environment.

As displayed in panel a (top graph) of Figure 4a, we apply a square wave voltage to the polymer films (3.5 cycles). Long-term measurements can be found in Figure S3 in the Supporting Information. The respective voltage is set for 15 s each. The measured normalized transmittance as function of time through both polymer films is depicted in the middle and bottom graphs in panel a. We observe fast and fully reversible switching for both films. Already from these graphs one can see that the rise time (oxidation/doping) is much faster for the PEDOT:PSS film which was *not* heat-treated (green curve). One possible reason is that the PEDOT:PSS films still retain water when the film is only exposed to room temperature drying. It is well-known that PEDOT:PSS is very hygroscopic and easily swollen by remaining amounts of water [48, 49]. Some of the water might even enter the electrolyte which might increase ion mobility. Furthermore, the PEDOT:PSS morphology has to be considered as well [50, 51]. Typically, an interpenetrating continuous structure of PEDOT and PSS is assumed, whereas electronic charge transport is strongly influenced by the crystallinity of the PEDOT phase. In the case of heating the morphology will be different. Heat-treatment of semicrystalline conjugated polymers is well-known to increase the overall crystallinity within films [52] and even in liquid electrolytes the crystallinity has shown to strongly influence electrochemical doping and dedoping [47, 53]. One could assume that the switching times are faster in the non-heated PEDOT:PSS films, because the PEDOT:PSS is less crystalline and therefore the ions can better penetrate the bulk of the films. The non-heat-treated films probably have a more accessible porous structure which eases doping and dedoping and accompanying ion intercalation and expulsion within the PEDOT:PSS films at the interface with the polymer gel electrolyte. All these effects would increase the rate of ion transport in the polymer, overall speeding up the switching time in the non-treated case.

To analyze this finding in more detail, we plot in Figure 4b a zoom-in on the first rise and first fall curve. Additionally, we plot the curves for PEDOT:PSS films where the electrochemical device was air-sealed after assembly (red: heat-treated PEDOT:PSS, blue: not heat-treated PEDOT:PSS). The switching times (80% contrast change) are extracted from panel b and plotted in Figure 4c. We find also in the case of the air-sealed devices that the rise time is much faster for the non-treated PEDOT:PSS films (blue, 42 ms) in comparison to the heat-treated films (red, 115 ms). The fall times are identical with 57 ms. Most dominantly, there is a strong influence of the device sealing on the switching dynamics. Overall, the switching times of the air-sealed PEDOT:PSS films are significantly faster than the films where the device was not sealed. Possible reasons are a strong influence of oxygen as well as evaporation of acetonitrile from the gel-electrolyte in case of the non-sealed devices. The fastest switching dynamics are obtained for PEDOT:PSS films which were not heat-treated and where the device was air-sealed (blue). We reach with this combination a total duty cycle switching time of 99 ms, equivalent to possible switching frequencies of up to 10.1 Hz. The other combinations reach 1490 ms/0.7 Hz (orange), 506 ms/2.0 Hz (green), and 172 ms/5.8 Hz (red). It should be noted that, at this point, we do not know yet the exact dielectric function and thus the metallic wavelength range of the presented PEDOT:PSS films. This will be prospect of future work.

## 3 Conclusion

In conclusion, we have demonstrated a metadevice for beam switching which comprises a metasurface from PEDOT:PSS in combination with a PEO-based gel polymer electrolyte. We have shown that with this electrolyte the plasmonic resonance of the polymer nanoantennas can be switched ON and OFF with CMOS compatible voltages. Hereby, a gradual decrease of the amplitude of the plasmonic resonance was observed up to a fully insulating state with no remaining plasmonic modulation. Additionally, our beam switching metasurface showed 100% optical contrast in the diffracted beam over multiple switching cycles.

Our work will greatly boost the integration of polymer nanoantennas and metasurfaces into on-chip electro-optic devices. Especially for applications in AR, VR, and MR, the great benefit of polymer metasurfaces is the possibility that they can be actively operated in reflection *and* in transmission. This is particularly important for any miniaturized optical elements on smart glasses or contact lenses. What is more, the purely electrically switchable metal-to-insulator transition in our polymer allows for very high contrast optical devices and applications. Due to the very small lateral dimensions of polymer nanoantennas even sub-micrometer pixels might become feasible, necessary for future 3D holography. Finally, polymer material engineering can push the operation from the IR towards the visible spectral range by further increasing doping levels and charge carrier concentrations. Future adjustments to the electrolyte and its combination with the metallic polymer nanoantennas will promote even faster switching frequencies as well as less degradation [54]. Most dominantly, such adjustments can be the viscosity of the electrolyte as well as its exact composition. Adjustments to these parameters will change ion mobility and thus change the ion diffusion time constants, ultimately allowing to change the switching times of PEDOT:PSS.

## Supplementary Material

Supplementary Material Details
