# Supplementary material for: Electrically switchable metallic polymer metasurface device with gel polymer electrolyte
Source: Nanophotonics. 2023 Jan 24;12(8):1397–404. doi: 10.1515/nanoph-2022-0654 (PMC10125172; doi:10.1515/nanoph-2022-0654)
Supplement: Supplementary file 1 — Supplementary Material Details [file j_nanoph-2022-0654_suppl_001.docx]

**Supporting Information**

**for**

**Electrically switchable metallic polymer metasurface device with gel electrolyte**

*Derek de Jong^1,2,^*^†^*, Julian Karst^1,^*^†^*, Dominik Ludescher^1^, Moritz Floess^1^, Sophia Moell^1^, Klaus Dirnberger^3^, Mario Hentschel^1^, Sabine Ludwigs^3^, Paul V. Braun^2^, and Harald Giessen^1^*

^1^ 4th Physics Institute and Research Center SCoPE, University of Stuttgart, Pfaffenwaldring 57, 70569 Stuttgart, Germany

^2^ Department of Materials Science and Engineering, Materials Research Laboratory, and Beckman Institute for Advanced Science and Technology, University of Illinois Urbana-Champaign, Urbana, IL 61801, USA

^3^ IPOC-Functional Polymers, Institute of Polymer Chemistry, University of Stuttgart, Pfaffenwaldring 55, 70569 Stuttgart, Germany

^†^ These authors contributed equally

**
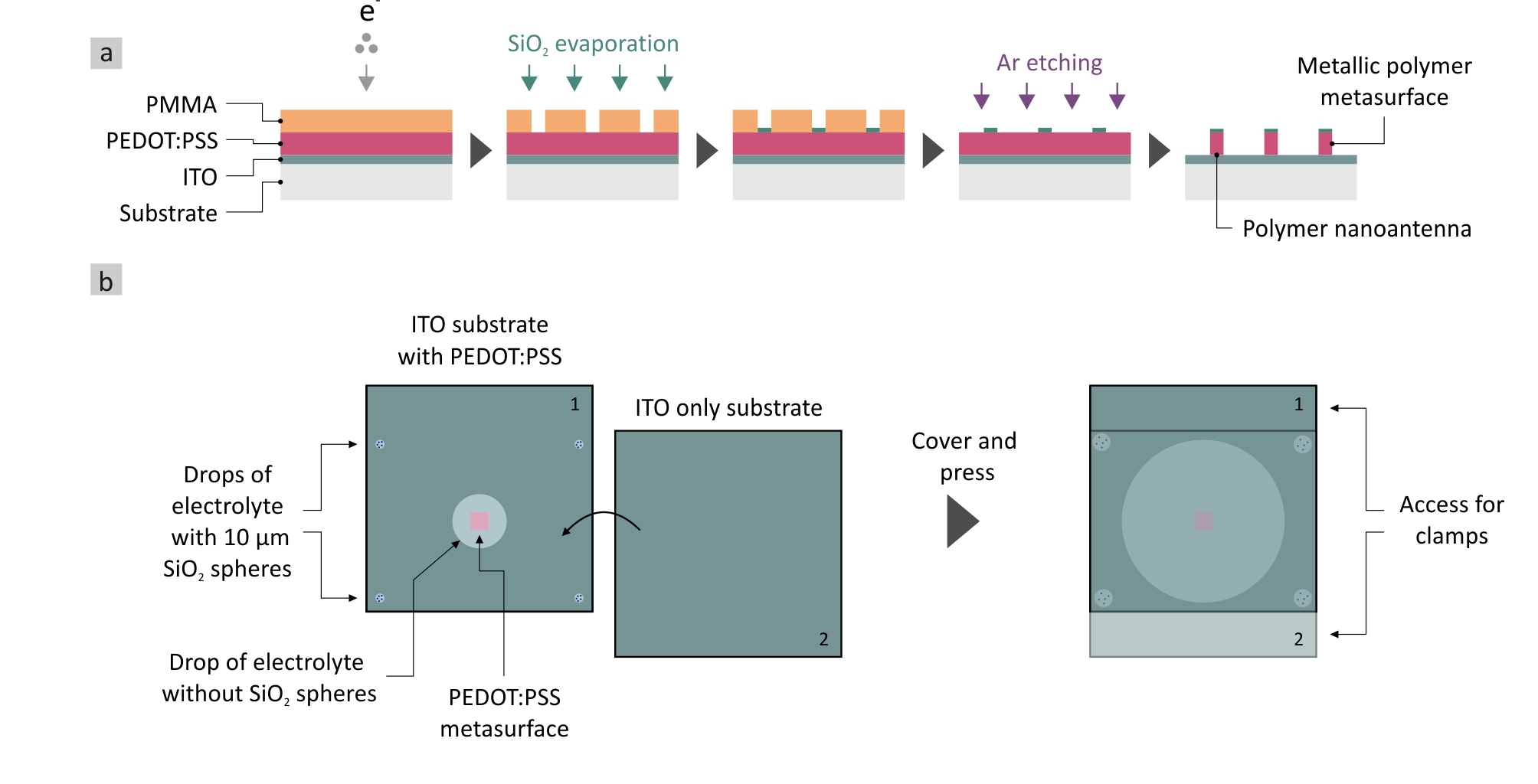
**

**Figure S1. Fabrication and assembly of metadevice.** (a) Electron beam lithography (EBL) to fabricate metasurface from metallic polymer PEDOT:PSS. A 90 nm thin film of PEDOT:PSS (Heraeus PH1000, Ossila) is spin-coated on an ITO-coated glass substrate and dried (120°C, 15 min). We use a double-layer PMMA positive tone resist (Allresist AR-P 642.06 200k, Allresist AR-P 672.02 950k) to define the nanoantennas with EBL. After development in methylisobutylketone (MIBK), we evaporate SiO_2_ followed by lift-off in acetone. The SiO_2_ antennas serve as etch mask for the subsequent argon (Ar) etching process. Finally, one obtains metallic polymer nanoantennas/metasurfaces from PEDOT:PSS on ITO. (b) Assembly of metadevice. Sample one comprises the PEDOT:PSS on the ITO substrate. Sample 2 is a plain ITO covered glass substrate, serving as the counter electrode and sealing of the device on the top. The gel polymer electrolyte preparation was as follows: PEO (100 kg/mol) was dissolved in acetonitrile (anhydrous, 99.8%) with a concentration of 0.4 g/ml. The ratio of PEO to conducting salt LiClO_4_ was 8:1 per weight. All chemicals were purchased from Sigma Aldrich and used as received. We put 4 drops of electrolyte with 10 µm SiO_2_ spheres on the edges of sample 1. The metasurface itself is covered with a drop of gel polymer electrolyte without the spheres. When putting both samples together and pressing them onto each other the electrolyte will spread out. The SiO_2_ spheres at the edges will act as distance control between both ITO samples as they cannot be compressed. Such, the electrolyte has a well-defined thickness of 10 µm. Optionally, the device is air-sealed with UV glue on the edges to prevent exposure to oxygen.

**
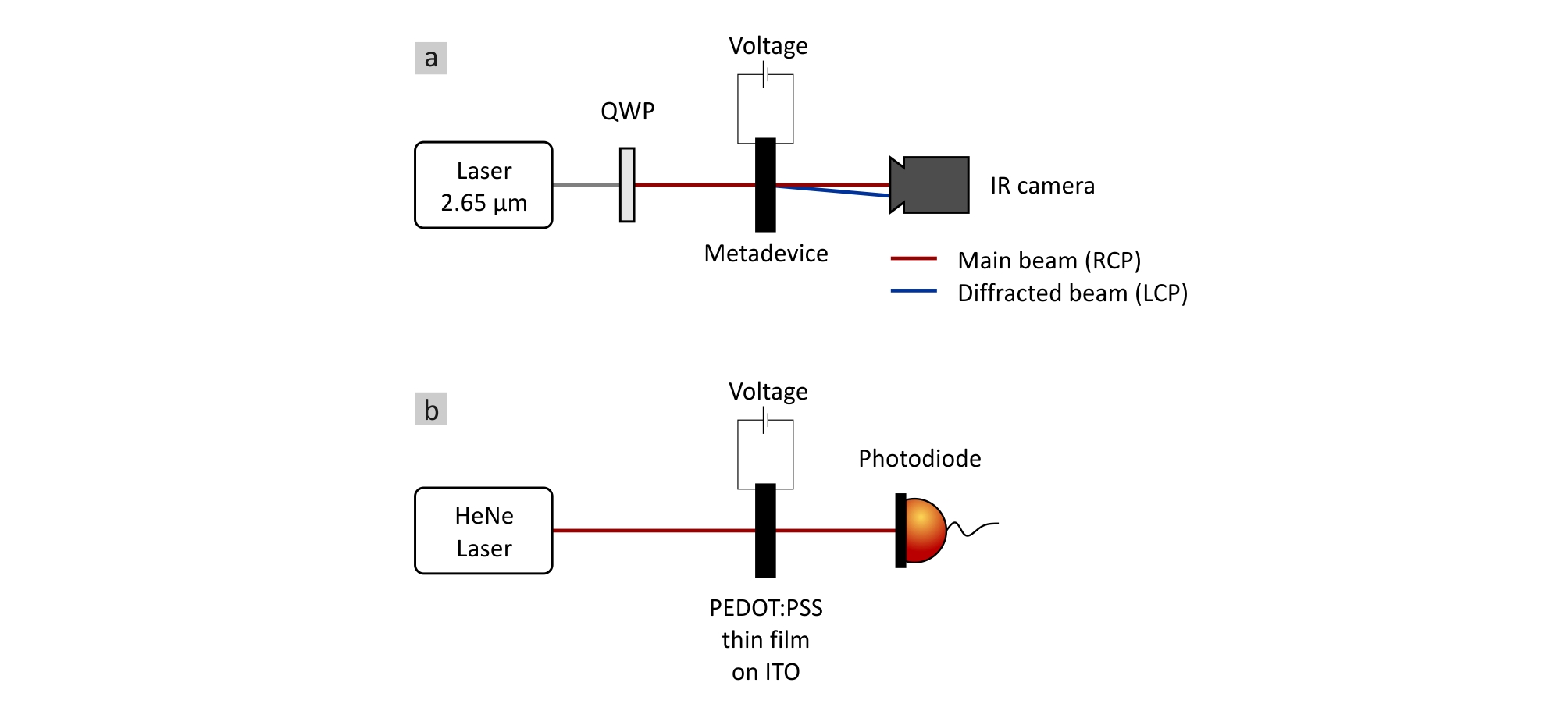
**

**Figure S2. Measurement setups.** (a) Simplistic sketch of setup to measure beam switching as displayed in Figure 3 in the main manuscript. An IR laser (Stuttgart Instruments Alpha HP) is tuned to 2.65 µm illumination wavelength. A quarter waveplate (QWP) creates right circularly polarized light (RCP) which impinges on the beam switching metadevice. The state of the metadevice is controlled by the external applied voltage. An IR camera detects main transmitted beam as well as the diffracted beam, which possesses opposite helicity (LCP) as the metasurface acts as a polarization converter. (b) Setup to measure the switching times as depicted in Figure 4 in the main manuscript. We use a helium neon (HeNe) laser to illuminate the PEDOT:PSS thin films on ITO. The transmitted intensity through the film is measured with a photodiode while the applied voltage is switched.

**
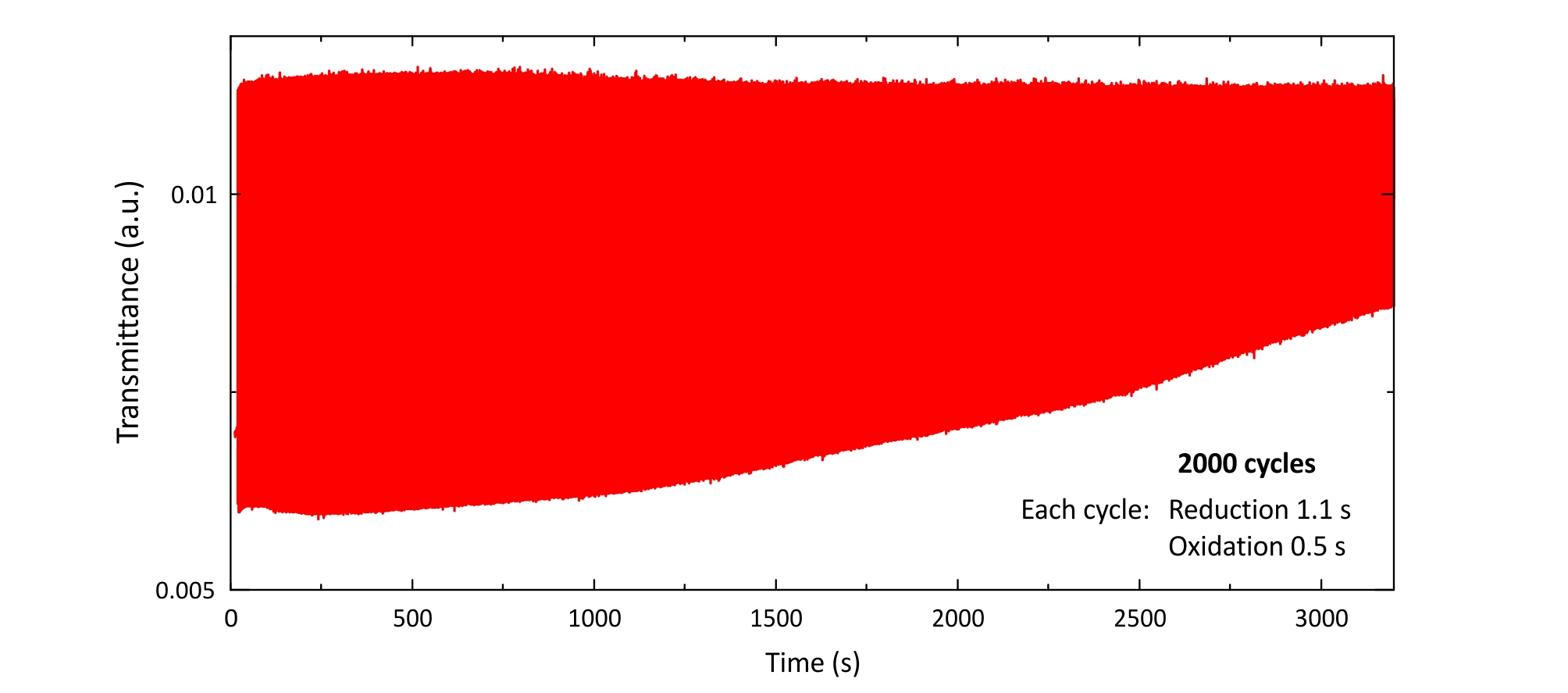
**

**Figure S3. Long term measurement on electrical switching of non-sealed, heat-treated PEDOT:PSS.** Transmittance through a 90 nm PEDOT:PSS thin film as function of time for 2000 cycles. The negative voltage (reduction, insulating) was applied for 1.1 s, the positive voltage (oxidation, metallic) for 0.5 s. The initial transmittance modulation between the two states is 5.43 × 10^-3^, whereas after 2000 cycles is decreases to 2.75 × 10^-3^. This means that the PEDOT:PSS has degraded by 50% in its optical response.
